# Measuring spatial accessibility and within-province disparities in accessibility to county hospitals in Shaanxi Province of Western China based on web mapping navigation data

**DOI:** 10.1186/s12939-020-01217-0

**Published:** 2020-06-18

**Authors:** Chi Shen, Zhongliang Zhou, Sha Lai, Li Lu, Wanyue Dong, Min Su, Jian Zhang, Xinyu Wang, Qiwei Deng, Yaru Chen, Xi Chen

**Affiliations:** 1 School of Public Policy and Administration, Xi’an Jiaotong University, No.28 Xianning West Road, Xi’an, 710049 Shaanxi China; 2 Team IETO, Bordeaux Population Health Research Center, University de Bordeaux, 33076 Bordeaux, France; 3 School of Public Health, Health Science Center, Xi’an Jiaotong University, Xi’an, 710063 China; 4 School of Public Administration, Inner Mongolia University, Hohhot, 010021 China; 5 Centre for HealthCare Innovation Research, Cass Business School & School of Health Sciences, City, University of London, London, EC1V 0HB UK; 6 Department of Health Policy and Management, Yale School of Public Health, New Haven, CT 06520 USA

**Keywords:** Spatial accessibility, County hospitals, Web crawler, Navigation

## Abstract

**Background:**

The Chinese government proposed the “XIAO BING BU CHU CUN, DA BING BU CHU XIAN” initiative in 2016, which states the rate of health care service provided by county hospitals should reach 90% of overall health care service provision. The prerequisite for achieving this goal is that citizens should be able to access county hospitals’ services conveniently and impartially. However, little research has been done on the actual levels of the spatial accessibility of citizens to county hospitals in Western China. Therefore, we aimed to measure the spatial accessibility to county hospitals for county residents and to identify any regional disparities in Shaanxi Province in Western China.

**Methods:**

We implemented a novel method – involving utilizing navigation data from the AutoNavi web mapping system (knows as Gaode map in Chinese) – to assess the time and distance from villages and neighborhoods to the county hospitals. The navigation data were collected by request through an application-programming-interface using a web crawler (web data extraction tool) in Python. The shortest driving time and distance were extracted from the navigation data. The travel impedance to the nearest provider (TINP) indicator was used to measure spatial accessibility.

**Results:**

The results show that county residents in Western China’s Shaanxi Province have poor spatial accessibility to county hospitals. Only 68.8% of villages and neighborhoods are within 60 min travel time (based on driving mode) to a county hospital, while 13.4% of such villages and neighborhoods are beyond 90 min travel time. Moreover, a significant within-province disparity exists, with residents in the central area enjoying the best accessibility to county hospitals, while the northern and southern areas still need improvements in accessibility.

**Conclusions:**

Focused health resource planning is required to improve the spatial accessibility to county hospitals and to eliminate regional disparities. Further studies are called for to integrate the navigation data of web mapping systems with GIS methods to the measure spatial accessibility of health facilities in more complex contexts.

## Introduction

The Chinese government issued an outline of the “Healthy China 2030” plan on October 25, 2016, which aims to improve people’s health and re-emphasizes the need to “provide an equal and accessible, systematic and sustainable health service” [1].

Equity and accessibility have become two of the most important goals of China’s health system. Equity and accessibility mean there should be no differences between urban and rural areas, different regions, and populations in terms of health service utilization, health outcomes, and access to health resources^1^ [5]. Here, accessibility generally refers to spatial accessibility (the convenience to reach a health care institution) and economic accessibility (whether the health care is affordable or not) in China [6]. Spatial accessibility not only measures the utilization of health services, but also can affect residents’ health status and health service needs [7, 8]. Measurement of the spatial accessibility of health resources may provide effective evidence for the need for health resource reallocation and improved regional health planning.

Existing studies on the spatial accessibility of health resources in China can be mainly summarized into two types based on their focus. First, studies have assessed the spatial accessibility of residents to different types of health institutions from the perspective of health system research. These studies have revealed that the distribution of hospital beds at the county level is highly spatially clustered [9]. Clear gaps in spatial access to primary health care were found within Sichuan Province in China [10], where 69% of villages have lower spatial accessibility to health services compared with the average county level in Jiangsu Province [11], while the spatial accessibility of public hospitals in Beijing was improved by referral reform in 2015, which also increased the inequality of access to medical resources between towns and streets [12]. Second, some studies have implemented various methods to evaluate the feasibility of using these methods to measure special accessibility, such as the “two-step optimization for spatial accessibility improvement” has been verified can balance the dual goals of efficiency and equality by combining the two steps in a true hybrid optimization model [13], and the two-steps floating catchment area method was verified to be able to reveal detailed spatial distribution differences in larger areas (such as cities) [14]. In conclusion, the spatial accessibility of health resources is a popular research fieldin China, with researchers tending to measure spatial accessibility from the perspective of health systems and methodologies. However, studies from the perspective of health systems are not enough, especially in terms of county-level hospitals.

China’s health care delivery system shows an urban-rural dual structure, with the three-tier health care delivery system playing the most important role in providing accessible and sustainable basic health services in rural areas of China [1, 10]. County-level hospitals are the highest level of health care institutions in the county regions, and therefore appropriate accessibility to county-level hospitals is an important prerequisite for supporting the health needs of rural residents [15]. The Chinese government proposed the “XIAO BING BU CHU CUN, DA BING BU CHU XIAN” initiative in 2016, which means the rate of health care service provided by county hospitals should reach 90% of overall health care service provision [16]. The prerequisite for achieving this goal is that residents should be able to access health care services from county hospitals conveniently and impartially. However, there is a lack of research documenting the level of spatial accessibility of county hospitals in China.

The commonly used methods to measure residents’ spatial accessibility to public services are provider-to-population ratios (PPR), nearest-neighbor analysis (NNA), and two-step floating catchment area (2SFCA), as well as a series of modified methods [2, 10, 17–19]. There are two main problems in the measurement of spatial accessibility: i) identification of the population distribution, and ii) accurate calculation of the time and distance between residential areas and the health care institutions serving them. The most widely used solutions to obtain population distribution include 1) using population census [8, 11, 20–22], or 2) using a GIS package to cut the map into grid cells with different areas and then to evenly assign the total population of the area to each grid unit and treat its center as the population distribution coordinate point [7, 9, 23]. The most common method to measure time and distance between points is to use road networks in the GIS package together with information on the applicable speed limit criteria on those road networks to measure travel time [7, 9, 23]. Some limitations of this approach include that the traditional census is conducted at intervals and therefore includes hysteresis, that errors can occur from cutting up the map, and that using road network maps can lead to rough estimates.

Therefore, this study aimed to measure spatial accessibility to county hospitals and any disparities in accessibility for county residents in 73 administrative counties in Shaanxi Province of Western China. We first used the navigation data of online digital maps to assess the time and distance from residents to county hospitals in Shaanxi Province. The navigation data were collected from AutoNavi, a Chinese web mapping, navigation, and location-based services provider, via our request to open an application programming interface (API) using web crawler technology.

### Data collection and methods

#### Research area

Shaanxi Province is the most developed province in Western China, with an area of 205,800 km^2^ and a total population of 38.35 million in 2017 [24]. Geographically, the central, southern, and northern regions of Shaanxi Province differ significantly. The central part is on a plain and includes the wealthiest area in Shaanxi Province, while the southern part includes the Qinling Mountains, and the northern part covers the Loess Plateau. The economy is less developed with a relatively small population density in the southern and northern parts. This study divides Shaanxi Province into three regions based on geographic and economic conditions.

#### Data collection scheme

In order to measure the spatial accessibility to health resources, three types of data were basically needed: geographical distribution of the population, the geographical location of hospitals, and the time and distance between residents and the hospitals. Therefore, we collected data in three steps.

First, considering the uneven distribution of the population, we used the geographical location of the villages and neighborhoods to identify the population distribution. Here, two strategies were adopted in our study:

i) For villages and neighborhoods with a village clinic, we selected the coordinates of the village clinics to represent the population distribution since the village clinics should be in an area with relatively high concentration of the village population to cover the population of the village to the greatest extent.
ii) For villages and neighborhoods whose village clinic we could not acquire or that have multiple village clinics, we selected the default coordinates provided by the web mapping navigation service provider. This coordinate usually defaults to the location of the village or neighborhood office, which is usually located in a populated area.

Second, we obtained the names of the county hospitals from the Health Commission of Shaanxi Province, and then we directly used the names of the hospitals to get their geographical locations from the web map.

Third, the time and distance between each village and neighborhood to the county hospitals were collected from the navigation results provided bythe web mapping navigation service. We chose the fastest route, but not the highway route (because China’s highway import and export are usually set around the county), to get the time and distance from villages and neighborhoods to the local county hospital by using the real-time navigation data of the AutoNavi map in the driving mode. The reason why only the local county hospitals were selected is that the Chinese new rural cooperative medical insurance implemented in rural areas provides cover at the county level only. In this study, we assumed that due to the medical insurance reimbursement strategy, residents were less likely to visit a doctor in another county-level hospital outside their own county [11].

#### Data collection method

To perform the data collection, first, we obtained the names of the village clinics and county hospitals in overall Shaanxi Province from the Shaanxi Provincial Health Statistics Annual Report in 2017, which was provided by the Health Commission of Shaanxi Province. In addition, we also obtained the names of the village and neighborhood committees in overall Shaanxi Province from the website of the National Bureau of Statistics [25].

Second, we used the geocoding interface of AutoNavi map to collect the coordinates of all the villages and neighborhoods and county hospitals. The requests through the API for the geocoding of the AutoNavi map were conducted by using a web crawler (a web data extraction tool) in the Python 3.6 program [26]. The URL of this geocoding interface can be found in the footnote below.^2^ AutoNavi map, known as Gaode in Chinese, was founded in 2011 and is one of the largest web mapping, navigation, and location-based services providers in China. It offers map services at Amap.com and on a mobile App too.

**Fig. 1:**
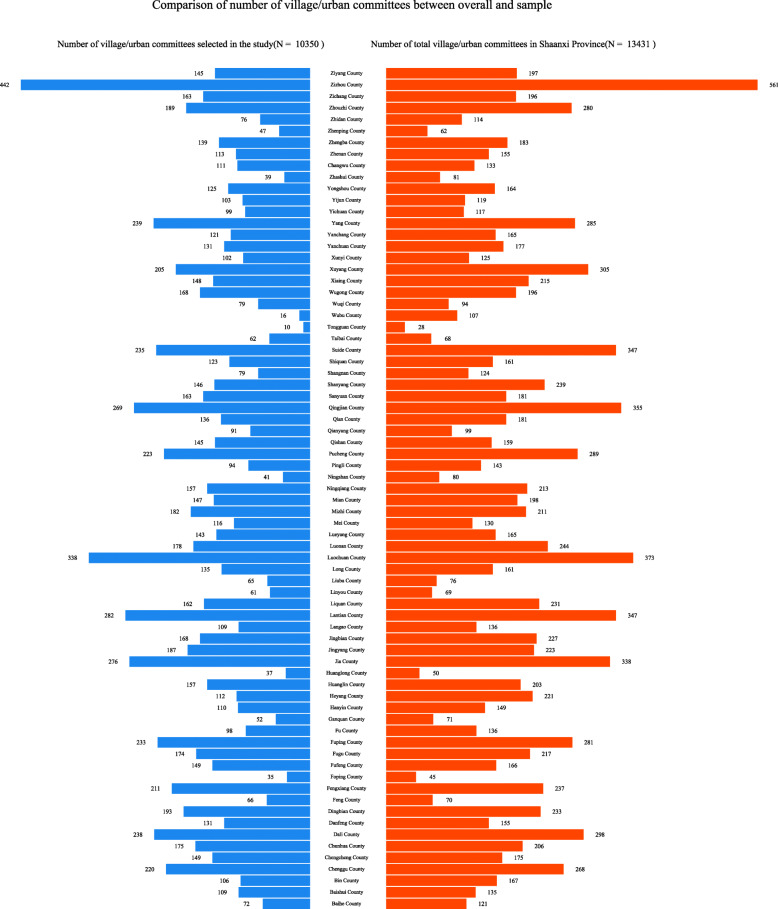
Sample information about villages and neighborhoods included the present study

Third, navigation data, including driving time and distance, were collected by using the path planning interface by setting the coordinates of the villages and neighborhoods as the starting point and the coordinates of a county hospital in the district as the endpoint. The URL of the path planning interface can be found in the footnote below.^3^ To consider the influence of the traffic conditions at different times, this study was performed four times randomly: the morning (10:00 to 11:00) and afternoon (14:00 to 15:00) on November 23, 2018 (Friday) and November 27, 2018 (Tuesday). For the time periods studied, crawling requests were made by Python to the AutoNavi mapping for the four time periods, and we took the average value of the results for the four time periods. Finally, data on 10,350 villages and neighborhoods (total of 13,074 villages and neighborhoods) from 73 counties of Shaanxi Province were obtained in our study (Fig. 1).

#### Analysis methods

The travel impedance to a nearest provider (TINP) was used to evaluate the spatial accessibility in this study. Here, TINP measured the spatial accessibility by using indicators such as the distance, time, or cost from the place of residence to the nearest medical institution, expressed in terms of a straight Euclidean distance (straight line) [2]. Distance and time were chosem as they are indicators that directly reflect spatial accessibility, whereby the closer the distance, the shorter the travel time and the higher the accessibility. Although TINP ignores the supply of health resources, this method is applicable to situations where the choice of seeking health care service is relatively simple, as in rural areas. In addition, we used more precise traffic distance downloads from a web map instead of the Euclidean distance in this study.

We calculated Getis-Ord Gi* statistics for the spatial association of each county to explore whether there were any disparities in spatial accessibility [27, 28]. The Gi* statistic returned for each county was recorded as a z-score [29]. A high positive z-score and small p-value for a county represent a spatial clustering of high values (hot spot)); whereas a low negative z-score and small p-value represent a spatial clustering of low values (cold spot). The higher or lower the z-score, the more intense the clustering. A z-score close to zero means no significant spatial clustering. Getis-Ord Gi* statistics were calculated by using the R package ‘spdep’ [30]. The spatial relationships of counties were defined as Queen’s Case. The distance and time of counties were recorded as the averages of distance and time of villages and neighborhoods.

We used concentration curve and the concentration index (CI), a method recommended by the World Bank to measure the inequality in health indicators related to living standards [31], to explore the influence of gross domestic product (GDP) and population on the differences in spatial accessibility to county hospital across counties.

In the concentration curve plot, the horizontal axis is the cumulative percentage of the observation unit (county in our study) ranked in ascending order by living standards (rank variable), and the vertical axis is the cumulative percentage of the health indicator. Originally, the living standard is a socioeconomic indicator, but we extended it to GDP and population. Concentration curve can be used to examine inequality in any health sector variable of interest [31], such as health resources and health services [32, 33].

The value of CI is the double area between the concentration curve and the line of equality (the 45-degree line); it is a negative (positive) value when the concentration curve lies above (below) the line of equality. The range of CI is between − 1 and 1, where zero means no rank variable-related inequality, a negative (positive) value means a disproportionate concentration of the health indicator among the observation unit with the lower (higher) value in the rank variable. Generally, the calculation method of the concentration index is as follows:

$$ C=\frac{2}{\mu}\mathit{\operatorname{cov}}\left(h,r\right) $$

where, *h* is the health indicator, *μ* is its mean, and *r* is the fractional rank of the observation unit. In this study, we selected the average shortest time of the village/neighborhoods to county hospitals as the health indicator, GDP and population^4^ as the rank variables, and county as the observation unit.

## Results

### Level of spatial accessibility

**Fig. 2:**
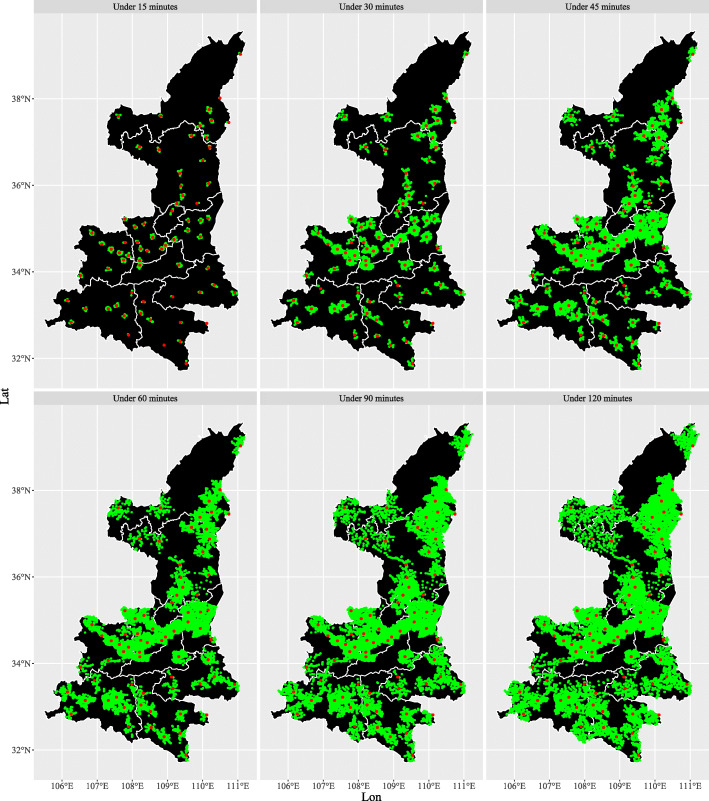
The distribution of villages and neighborhoods that can reach county hospitals in under different times in Shaanxi Province

From the perspective of the villages and neighborhoods level, the average driving distance from the villages and neighborhoods to the county hospitals was 28.4 km, with an average shortest travel time of 49.7 min. Only 68.9% of villages and neighborhoods are within 60 min travel time to reach county hospitals, while 13.4% would require more than 90 min to reach the county hospital (Table 2 and Fig. 2).

**Fig. 3:**
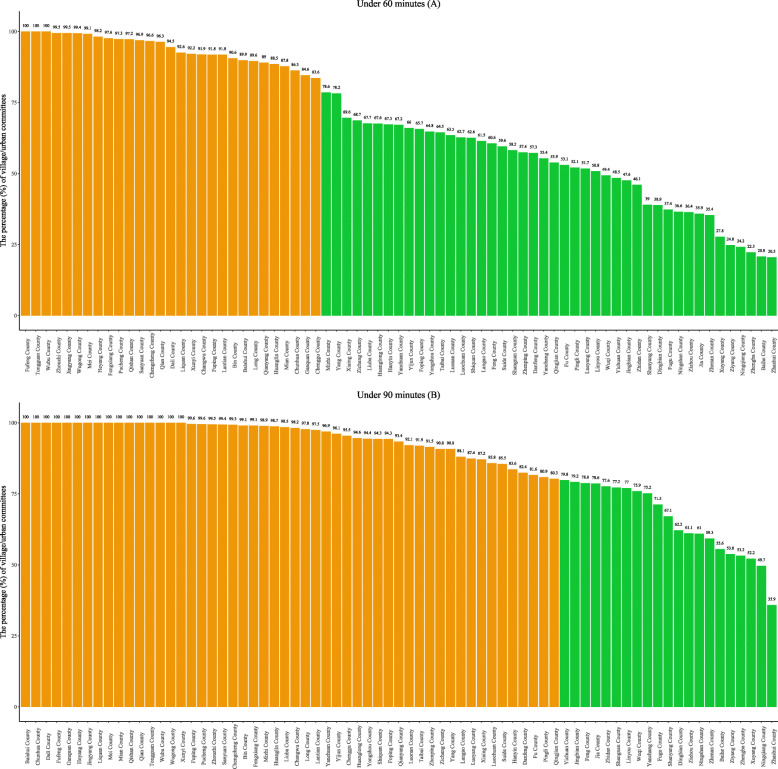
The percentage of villages and neighborhoods that can reach the county hospital in under 60 or 90 min per county in Shaanxi Province

From the perspective of the county level, we calculated the percentage of villages and neighborhoods where residents could reach county hospitals within 60 min and 90 min per county. Figure 3 shows that the number of the county where 100% of villages and neighborhoods can access to the county hospitals within 60 min and 90 min are 3 and 16, respectively. Therefore, we assumed that the standard measure was one where 80% of the villages and neighborhoods were within that travel time limit to reach a county hospital. We further calculated the frequency and percentage of counties in which 80% of villages or neighborhoods could access a county hospital within 60 min and 90 min, respectively, and found that only 39.7 and 71.2% of the counties in Shaanxi Province meet that standard (Table 2).

### Disparity of spatial accessibility

**Fig. 4:**
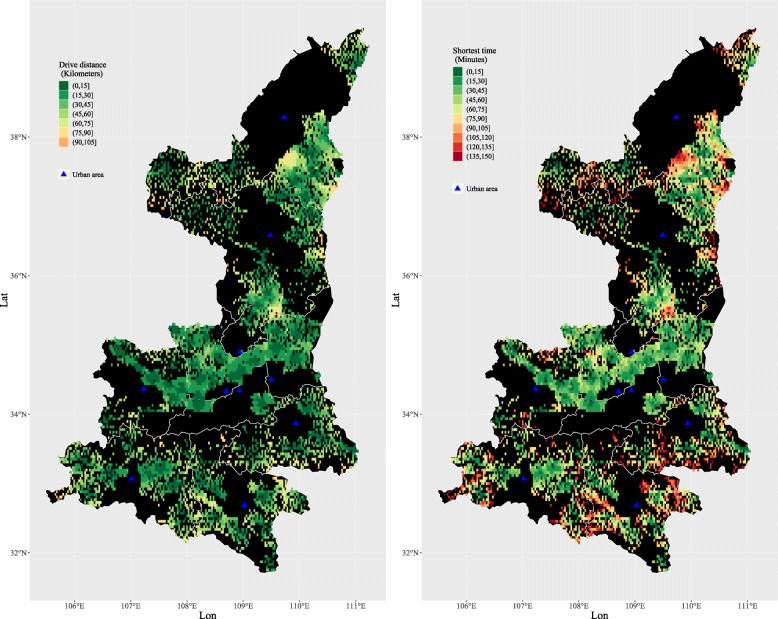
Regional disparities in the shortest driving time and distance within Shaanxi Province

**Fig. 5:**
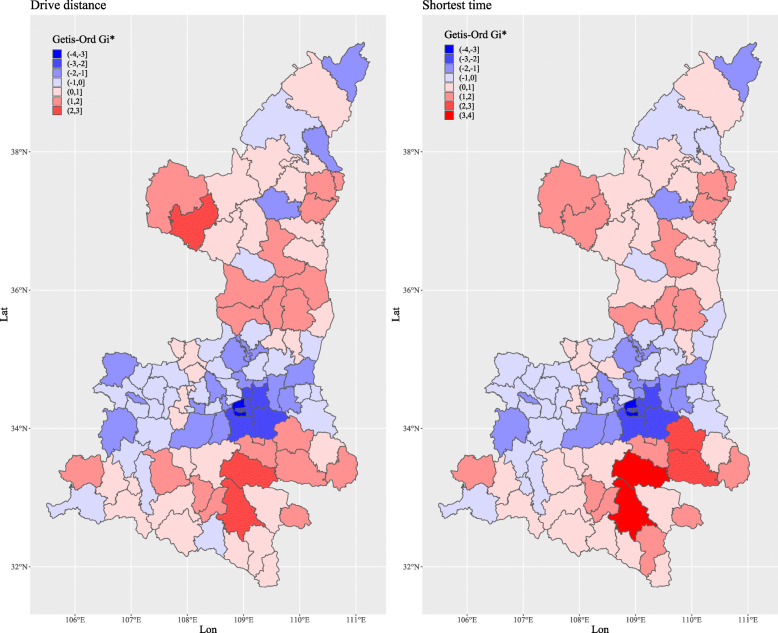
Getis-Ord Gi* statistics of the shortest driving time and distance at the county-level within Shaanxi Province

In terms of sub-regions, there is a large disparity of spatial accessibility in the central, northern, and southern regions of Shaanxi Province (Fig. 4). Meanwhile, we summarized the travel distances and times for the individual villages and neighborhoods into county-level values, where the values of each county are the averages of local villages and neighborhoods. Getis-Ord Gi* statistics show that the northern and southern regions of Shaanxi Province are hot areas, while the central region represents a cold area (Fig. 5), which means longer travel distances and times are clustered in the northern and southern regions. In other words, the worst spatial accessibility areas are clustered in the northern and southern regions. The central region has the best spatial accessibility due to its location in the plain and it incorporating richer areas, with an average driving distance from residential areas to county hospitals of 19.2 km and with the shortest average journey times of 33.5 min, and hence 91.6% of the residents could reach a county hospital within 1 h (Table 1). The proportions of residents that can arrive at the county hospitals within one hour in the northern and southern regions were 54.0 and 53.6%, respectively (Table 1). At the county level, the proportions where 80% of the villages and neighborhoods in the counties were within 60 min travel time to a county hospital were 82.8% in the central region, 15.0% in the northern region, and 8.3% in southern region. The figures based on arrival within 90 min were only 55.0% in the northern region and 58.3% in southern region (Table 2).

**Table 1:** Spatial accessibility to county hospitals at the villages and neighborhoods level

	Central Shaanxi	Northern Shaanxi	Southern Shaanxi	Overall	*F*	*p*
N	4119	3306	2925	10,350		
Distance(km), mean(sd)	19.2 (12.1)	35.2 (21.0)	33.8 (20.2)	28.4 (19.3)	935.7	< 0.001
Shortest time(minutes), mean(sd)	33.5 (19.3)	59.5 (32.7)	61.5 (35.5)	49.7 (31.9)	1060	< 0.001
Time range, %(*CI* 95%):						
Under 15 min	13.8 (12.8–14.9)	7.6 (6.7–8.5)	6.6 (5.7–7.5)	9.8 (9.2–10.4)		
Under 30 min	48.7 (47.1–50.2)	21.6 (20.2–23)	22.6 (21.1–24.1)	32.6 (31.7–33.6)		
Under 45 min	79 (77.7–80.2)	38.3 (36.6–39.9)	38.9 (37.2–40.7)	54.7 (53.7–55.6)		
Under 60 min	91.6 (90.8–92.4)	54.0 (52.3–55.7)	53.6 (51.8–55.4)	68.9 (68.0–69.8)		
Under 90 min	98.3 (97.9–98.7)	80.2 (78.9–81.6)	77.3 (75.8–78.9)	86.6 (86.0–87.3)		
Under 120 min	99.6 (99.4–99.8)	95.7 (95.0–96.4)	91.9 (90.9–92.9)	96.2 (95.8–96.5)

**Table 2:** Spatial accessibility to county hospitals at the county level

Areas	The number (%) of counties in which 80% of villages or neighborhoods can access a county hospital	
	Under 60 min	Under 90 min
Overall Shaanxi (N = 73)	29 (39.7%)	52 (71.2%)
Central Shaanxi (N = 29)	24 (82.8%)	27 (93.1%)
Northern Shaanxi (N = 20)	3 (15.0%)	11 (55.0%)
Southern Shaanxi (N = 24)	2 (8.3%)	14 (58.3%)

### Factors influencing the disparities in spatial accessibility

**Fig. 6:**
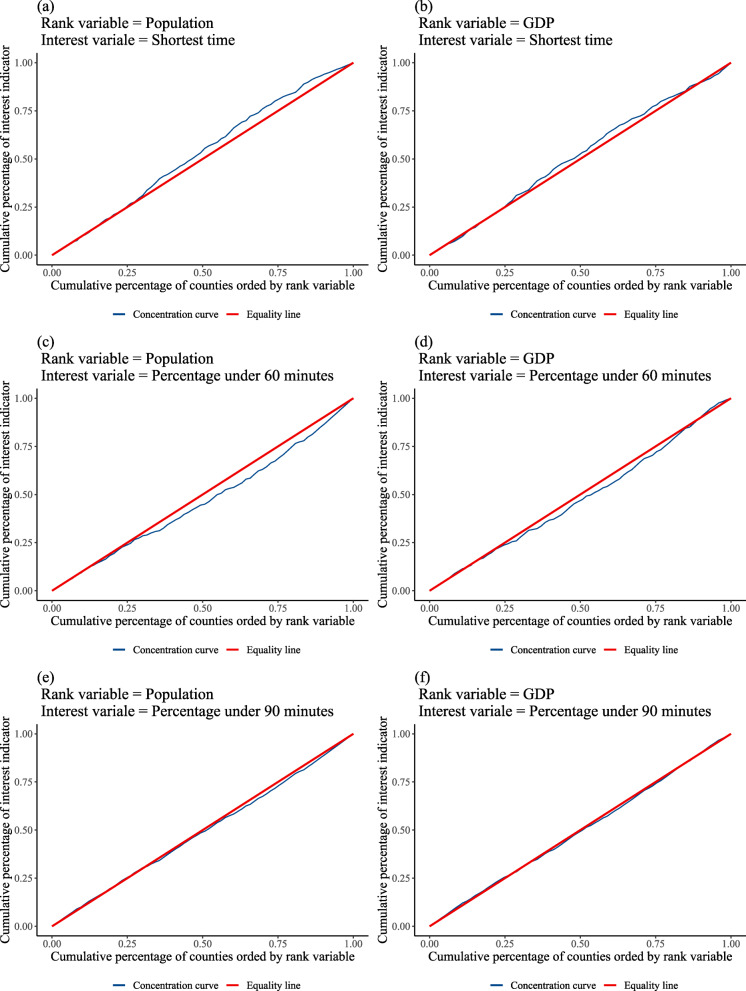
Concentration curves of the spatial accessibility to the county hospital of counties in Shaanxi Province. Note: ^a^ means the average shortest time for villages/neighborhoods in a county, ^b^ means the percentage of villages/neighborhoods that can access the county hospital in under 60 min in a county, ^c^ means the percentage of villages/neighborhoods that can access the county hospital in under 90 min in a county

In order to analysis the factors influencing the disparity in spatial accessibility to county hospital across counties, we calculated the concentration index to evaluate whether the population or GDP in a county affect the spatial accessibility to county hospital. The results (Table 3 and Fig. 6) show that the CI for the average shortest time for the village/neighborhood to reach the county hospital in a county, as ranked by population, was − 0.059, which means that counties with smaller populations tend to have a longer travel time to reach the county hospital than counties with larger populations. The CI of the percentage of villages/neighborhoods that can access a county hospital in under 60 min in a county was 0.070, which means the county hospitals in the counties with larger populations can cover more residents than the county hospitals in the county with smaller populations. Overall, the residents in a county with a larger population have a higher spatial accessibility to county hospitals. It may seem that the residents in a county with a higher GDP would have higher spatial accessibility to county hospitals too, however, the insignificant p-values did not support us to confirm this finding.

**Table 3:** Concentration Index (CI) of spatial accessibility to the county hospitals of counties in Shaanxi Province

Interest variable	Rank Variable							
	Population				GDP			
	N	CI	Std. Err.	p value	N	CI	Std. Err.	*p* value
Shortest time ^a^	73	−0.059	0.024	0.015	73	−0.030	0.025	0.227
Percentage under 60 min ^b^	73	0.070	0.023	0.003	73	0.037	0.024	0.119
Percentage under 90 min ^c^	73	0.016	0.012	0.183	73	0.006	0.012	0.657

^a^ means the average shortest time for villages/neighborhoods in a county, ^b^ means the percentage of villages/neighborhoods that can access the county hospital in under 60 min in a county, ^c^ means the percentage of villages/neighborhoods that can access the county hospital in under 90 min in a county

## Discussion

There are three key findings from our study. First, residents in county areas in Shaanxi Province have poor spatial accessibility to county hospitals, whereby only 68.8% of villages and neighborhoods are within 60 min travel time to county hospitals, while 13.4% still need more than 90 min for residents to reach the county hospital. Second, there is a large disparity within Shaanxi Province, whereby the residents in the central area have the best spatial accessibility to county hospitals, while the northern and southern area still need improvement in the accessibility. Third, residents in counties with a larger population have a higher spatial accessibility to county hospitals. Moreover, our study proved that using the navigation data of a web map to measure spatial accessibility to health resources is a feasible technique.

Our study shows that Shaanxi Province has a low-level spatial accessibility to county-level hospitals. In fact, we think the spatial accessibility might be even worse, because we selected the driving mode to calculate the distance and time in the process of crawling the navigation data. The original intention of selecting the driving mode was to simplify the data collection process; however, in practice, there is a low ownership of moto vehicle pers. 100 urban households, only 29.7 in 2017 in China [34], which means China has not yet developed to the stage where every household has a vehicle, especially in the counties and rural areas. The main travel modes in China’s rural areas are motorcycles and e-bikes, which have a lower speed than vehicles, and therefore, the spatial accessibility in the county area may be overestimated in our study. However, this overestimation does not affect the comparisons made here of the spatial accessibility considered under a uniform travel mode, so comments on the disparity within Shaanxi Province are valid. On the other hand, since Shaanxi is the most developed province in Western China, we have reason to infer that other Western provinces (Gansu and Ningxia, etc.) with less developed economic levels and worse geographical environments may have worse spatial accessibility to county hospitals.

Good spatial accessibility is an important prerequisite for residents to use health care services in a timely manner, whereby a low-level spatial accessibility may cause serval problems. One research study reported that asthma mortality showed a significant trend with an increase in travel time to hospital, and the relative risk was increased by 1.07 times for each additional 10 min [35]. Mortality from other time-critical diseases, like stroke, may also experience the same risk. Another study showed that geographical obstacles were one of the four major factors limiting persons with disabilities being able to access primary health care [36]. Moreover, we found an obvious regional disparity in the spatial accessibility to county hospitals across Shaanxi Province, and the disparity was highly correlated with the county’s population size. This disparity should be taken seriously. Further regional disparities in spatial accessibility were also observed for primary health institutions in Sichuan Province and for public hospitals in Beijing City [10, 12]. Regional variations are also not limited to China and commonly exist in many parts of the world, and have been reported in the literature in various studies, including significant geographic disparities in access to primary stroke centers in the United States [37], in access to health care facilities between urban and suburban seniors in Montreal Island [38], and in access to community resources between urban and rural areas of New Zealand [39]. It is well known that spatial accessibility influences health services utilization. Strong evidence supports that there is a strong pro-rich inequality in maternal health services and inpatient utilization in rural Western China [40, 41]. Accordingly, we have reasons to infer that the disparity in spatial accessibility may cause or aggravate the pro-rich inequality of health services utilization in Western China. Therefore, China should also pay attention to the equity of spatial accessibility to health care, although this may be something the Chinese government is aware of as it has already advocated that residents in every region should have equal access to health care resources.

How to improve the spatial accessibility to county hospitals in rural areas? Some cities in China have begun to set a goal of “15-minute health circle” for primary health care services [42]. Our study identified that it would be reasonable to set the residents’ spatial accessibility to county hospitals as “1 h” in Western China. In addition, the most direct approach to improve spatial accessibility is to improve transportation facilities in rural areas, but spatial accessibility should not be limited to just making improvements considering the physical distance. By establishing video telemedicine and a timely drug distribution system by using Internet technology to strengthen the flow of superior resources between the three-level (county–township–village) service networks, the spatial distance between residential areas and health care services in county hospitals could also be effectively improved.

Finally, one previous study analyzed the time and space accessibility of urban parks by using the path planning API provided by Gaode map [43], as we used in this study, which confirmed the feasibility of using navigation data of the web map to conduct spatial accessibility research. There are two advantages of this method: i) The accuracy of the data extracted from the navigation system is significantly higher than that roughly estimated by using road network maps. However, the measurement accuracy is unsatisfactory whether using the linear Euclidean distance or road network traffic distance. An accurate method to measure distance is very important since the measurement accuracy of the distance directly affects the accuracy of the assessment of spatial accessibility. ii) Navigation data provided by the web mapping realizing real-time updates, i.e., considering the road traffic situation, is more in line with the actual situation. In summary, combining the navigation data from a web map and two-step floating catchment area modified methods to measure spatial accessibility of health resources is highly feasible and has good application prospects.

We still can not avoid there being some limitations in this study, which we note here. First, since it is very difficult to collect specific population data at the village and neighborhood level, only the relatively simple nearest distance method was used to evaluate the spatial accessibility, which ignored the supply and demand of health resources. Second, distance and time data were only extracted under the driving mode, and the figures were not analyzed for using public transportation or other modes, which may not be consistent with the actual mode of travel of rural Chinese residents. Last, not all villages and neighborhoods in Shaanxi Province were involved because a small amount of data were unobtainable. Looking to the future, we are looking for a feasible method to collect the population data in villages and neighborhoods, fortunately, China is doing its sixth national population census, which will provide a chance to obtain accurate population data in villages and neighborhoods. At that time, we can consider the population and health resources for measuring spatial accessibility by combining the navigation data of the web map.

## Conclusion

We found that county residents in Western China’s provinces, as represented by Shaanxi Province, have a lower spatial accessibility to county hospitals, and significant regional disparities exist within provinces. Health policy and health resource planning are needed to improve the spatial accessibility and to eliminate regional disparity. Moreover, our study demonstrates the feasibility of using navigation data provided by a web map to measure spatial accessibility to health resources. Further research is needed to verify whether this new method is more accurate than using GIS for evaluation. We encourage further research to combine the navigation data of a web map with two-step floating catchment area modified methods to measure the spatial accessibility of health facilities in more complex situations.

## Data Availability

The data were collected from AutoNavi map, a Chinese web map and navigation service provider. This web map provides an open application programming interface for freely using data after registering as a developer. The application programming interface can be found here: (https://lbs.amap.com/api/webservice/guide/api/georegeo#geo) & (https://lbs.amap.com/api/webservice/guide/api/direction#driving).

## Abbreviations

API: Application Programming Interface
CI: Concentration Index
GDP: Gross Domestic Product
NNA: Nearest-Neighbor Analysis
PPR: Provider-to-Population Ratios
TINP: Travel Impedance to a Nearest Provider
2SFCA: Two-Step Floating Catchment Area

## Footnotes

1 Accessibility of health services has been widely studied and can be understood from different aspects, while there is no consistent on its definition and evaluation [2]. Penchansky and Thomas proposed that access to health services can be divided into five categories: availability, accessibility, accommodation, affordability, and acceptability [3]. These five aspects were considered to be the main influencing factors of health service utilization according to the behavioral model of Andersen’s health service utilization [4]. The access to health services was also assessed from four aspects: (1) affordability; (2) physical accessibility; (3) acceptability of services; and (4) not merely adequacy of supply [5].

2 https://lbs.amap.com/api/webservice/guide/api/georegeo#geo

3 https://lbs.amap.com/api/webservice/guide/api/direction#driving

4 Data were collected from the Shaanxi Statistical Yearbook in 2019. (http://tjj.shaanxi.gov.cn/upload/2020/pro/3sxtjnj/zk/indexch.htm)
